# Commentary: Somewhere between the root or no root argument: Selective sinus replacement

**DOI:** 10.1016/j.xjon.2020.12.019

**Published:** 2020-12-31

**Authors:** T. Brett Reece, Andrew L. Mesher, Muhammad Aftab

**Affiliations:** Division of Cardiothoracic Surgery, Department of Surgery, University of Colorado School of Medicine and University of Colorado Hospital, Aurora, Colo

**Figure:**
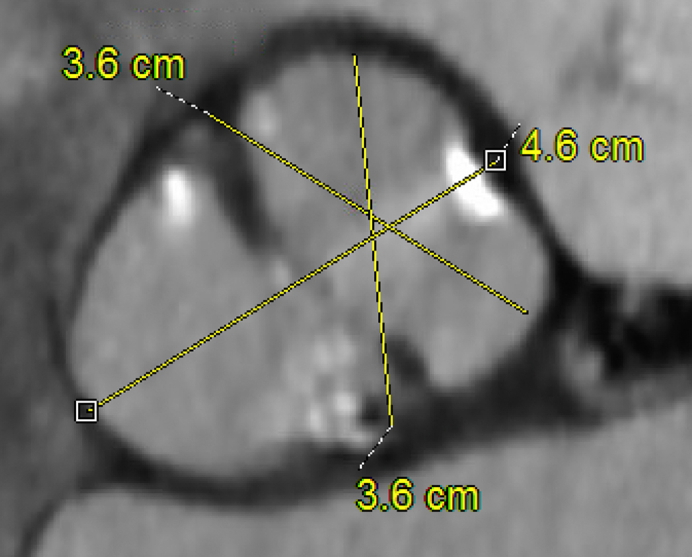
Sievers type 1 RL root with asymmetric dilatation may not require a full root replacement.

**Central Message**

The indications for bicuspid aortic root replacement are generally based on a single “root” measurement. However, bicuspid roots in particular can be particularly asymmetric, potentially requiring less than full root replacement.

See Article page 101.

Our colleagues from Galveston, led by Dr DeAnda, provide an excellent unbiased review of the indications for root replacement in bicuspid aortic valve patients undergoing valve and ascending aortic replacement surgery.^1^ The arguments expressed are based on the retrospective reviews of high-powered aortic programs. All these arguments base intervention on the bicuspid root by a singular root diameter; however, as the evolution of bicuspid aortic valve repair demonstrates, bicuspid phenotypes can vary immensely. The current guidelines do not address the ways in which asymmetry of the valve affect the asymmetry of the root with diverse sinus size. Essentially, one mid-sinus–to–commissure measurement does not adequately describe the entire root, so why would it mandate replacement of the entire root?

The authors makes a good point that root replacement can increase the potential risk of the procedure, most likely related to the coronary reimplantation. A less than full replacement may allow removal of the fragile tissue without manipulation one or both of the coronaries.

The asymmetric anatomy may facilitate a less risky partial root procedure. With the Sievers type 1 with right–left fusion phenotype accounting for >70% of the bicuspid aortic valves, the noncoronary sinus results in the largest sinus. If this alone is dilated, then single sinus replacement of the noncoronary sinus obviates the need to address the coronaries at all. The asymmetry of the aortic root may invalidate using a single maximal dimension, because the inferred LaPlacian wall stress and tension are not the same uniform distribution as in a tube or sphere. The resurgence of the root remodeling procedure has ushered in a wave of single sinus replacements, which mitigates the dilated sinus while avoiding potential issues with coronary manipulation. The literature on this approach is fairly limited, but discussion with several large programs suggests that the prevalence of this approach is probably underreported. Dr Lansac's group from Paris, who have revolutionized external aortic annular support, reported root stability in 18 patients at 4 years after replacement of the noncoronary sinus alone, as well as shorter bypass and cross-clamp times compared with full root remodeling.^2^ Jawitz and colleagues^3^ from Duke published a study of internal ring annuloplasty that included 13 patients with single sinus replacement and 2 patients with double sinus replacement with a combination of 2- and 3-leaflet valves. In short-term follow-up, no root dilation was identified. These are early examples of subtotal replacement that may serve a more significant role in bicuspid root management in the future.

In conclusion, the decision to replace the dilated root may be tailored to the specific root pathology. While a full root replacement eliminates the risk of future root issues, it also brings with it the risk of coronary reimplantation and manipulation. In cases where the root is asymmetrically dilated, the single sinus replacement could be a viable alternative to the previous binary options of root replacement or not.
